# The role of YAP1 in liver cancer stem cells: proven and potential mechanisms

**DOI:** 10.1186/s40364-022-00387-z

**Published:** 2022-06-07

**Authors:** Haofeng Wu, Yachong Liu, Zhibin Liao, Jie Mo, Qiaofeng Zhang, Bixiang Zhang, Lei Zhang

**Affiliations:** 1 Hepatic Surgery Center, Institute of Hepato-Pancreato-Bililary Surgery, Tongji Hospital, Tongji Medical College, Huazhong University of Science and Technology, Wuhan, 430030 People’s Republic of China; 2 Department of Hepatobiliary Surgery, Shanxi Bethune Hospital, Shanxi Academy of Medical Sciences, Shanxi Medical University; Shanxi Tongji Hospital, Tongji Medical College, Huazhong University of Science and Technology, Taiyuan, 030032 China

## Abstract

YAP1 (Yes-associated protein 1) is one of the principal factors that mediates oncogenesis by acting as a driver of gene expression. It has been confirmed to play an important role in organ volume control, stem cell function, tissue regeneration, tumorigenesis and tumor metastasis. Recent research findings show that YAP1 is correlated with the stemness of liver cancer stem cells, and liver cancer stem cells are closely associated with YAP1-induced tumor initiation and progression. This article reviews the advancements made in research on the mechanisms by which YAP1 promotes liver cancer stem cells and discusses some potential mechanisms that require further study.

## Background

Hepatocellular carcinoma (HCC) is one of the most aggressive malignancies, ranking as the third leading cause of cancer-associated mortality and the fifth most common cancer worldwide [1]. One of the reasons why liver cancer is prone to recurrence and aggression is cancer stem cells (CSCs) [2]. Cancer stem cells, also known as tumor-initiating cells (TICs), are a side population of cancer cells with stem cell features and the ability to self-renew and differentiate to generate heterogeneous cell populations. Studies have shown that liver cancer stem cells (LCSCs) exist in HCC and contribute to the tumor initiation, metastasis, and drug resistance of liver cancer [3]. To achieve a better understanding of LCSCs, various markers are used, such as the cell surface markers CD133, EpcAM, and ICAM-1 and the stem cell-associated genes OCT4, SOX2, and NANOG [4]. LCSCs with different surface markers may exhibit different characteristics [5]. For example, EpCAM-positive LCSCs show activation of Wingless (Wnt) signaling and resistance to sorafenib, while CD90-positive or CD105-positive LCSCs show resistance to 5-fluorouracil (5-FU) and transcatheter arterial chemoembolization (TACE) [5]. To determine whether CSC properties are enhanced, it is crucial to confirm the surface markers, stemness gene expression, sphere formation, tumorigenicity, and chemoresistance.

**Fig. 1:**
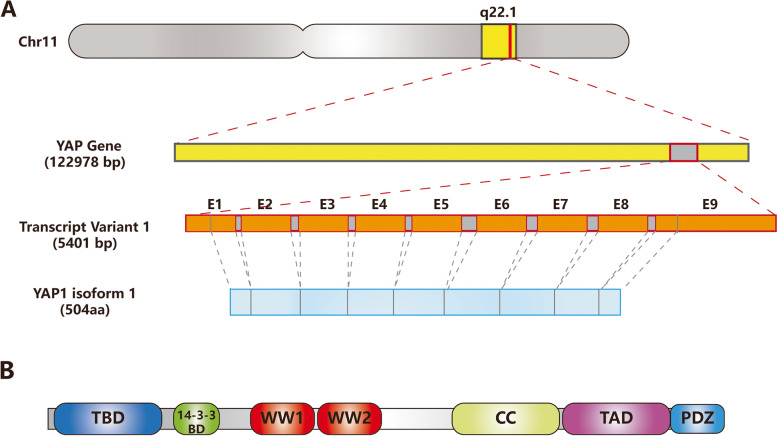
Schematic representation depicting the structure of YAP1 gene and protein. **A** YAP gene is located on 11q22.1. There are nine known isoforms of YAP, of which YAP1 isoform 1 (NM_001130145.3) is the most common transcript variant and contains 9 exons. **B** Schematic structure of human YAP1 proteins. TBD, TEAD-binding domain; WW, W-containing domain; CC, coiled-coil domain; TAD, transactivation domain

YAP1, a core component of the Hippo pathway with its paralog transcriptional coactivator with PDZ-binding motif (TAZ), acts as a cotranscriptional factor that translocates from the cytoplasm to the nucleus. The Hippo pathway is a highly conserved signaling pathway originally found in Drosophila [6] that plays a key role in regulating tissue and organ growth. The upstream components of YAP1 consist of the mammalian STE20-like protein (MST) 1/2, the large tumor suppressor homolog (LATS) 1/2 and their adaptor proteins mammalian ortholog of Salvator (WW45/Sav) and Mps One Binder Kinase Activator (MOBs). In the Hippo cascade, MST1/2 forms a complex with WW45 and phosphorylates LATS1/2 and MOBs. The activated LATS/MOB complex then phosphorylates YAP1 and results in its translocation into the cytoplasm and degradation [7]. Acting as a transcriptional coactivator, YAP1 interacts with TEA domain family member (TEAD) transcription factors to activate downstream gene expression. YAP1 protein consists of TEAD-binding domain (TBD), 14–3-3 binding domain, two W-containing domain (WW) domains, coiled-coil (CC) domain, transactivation domain (TAD), and PDZ domain. TBD interact with TEAD at three distinct interfaces. 14–3-3 binding domain includes S127 site, whose phosphorylation leads to binding with 14–3-3 protein. Then binding with 14–3-3 protein leads to cytosolic sequestration of YAP1 protein. Different from other domains with highly disorder, WW domains and CC domains are conserved domains. Studies have demonstrated that WW domains play an important role in the function of YAP1, but there are relatively few studies on the role of CC. TAD is essential in the transactivation, PDZ plays an important role in the nuclear translocation of YAP1 (Fig. 1) [8, 9]. In addition, YAP1/TAZ engage in crosstalk with other cancer-promoting pathways, such as the Notch pathway, MAPK pathway, and Wnt pathway [10]. YAP1 is closely correlated with epithelial–mesenchymal transition (EMT), stemness of cell, organ size, regeneration, and tumor progression [11]. YAP1 contributes to cancer development in several ways, including promoting malignant phenotypes, the expansion of cancer stem cells and drug resistance of cancer cells [12]. Studies have also found that YAP1 plays a crucial role in CSCs in lung cancer and prostate cancer [13].

Given that YAP1 is closely associated with liver cancer and CSCs, many studies have researched the interaction between LCSCs and YAP1. The expression of YAP1 is significantly increased in LCSCs, and the levels of YAP1 and YAP1-TEAD are positively correlated with the expression of stemness markers (NANOG, OCT-3/4, and CD133) [14] and the severity of HCC. Studies have found that YAP1-TEAD can induce the acquisition of liver cancer stemness, and YAP1-targeting treatment is effective for HCC with a cancer stem cell phenotype [15]. However, several mechanisms of YAP1-induced CSCs have not been demonstrated in liver cancer. In the present review, advances in the studies of the correlation between YAP1 and LCSCs, together with some factors that are not yet fully defined, will be presented from several aspects.

### Signaling pathway

**Fig. 2:**
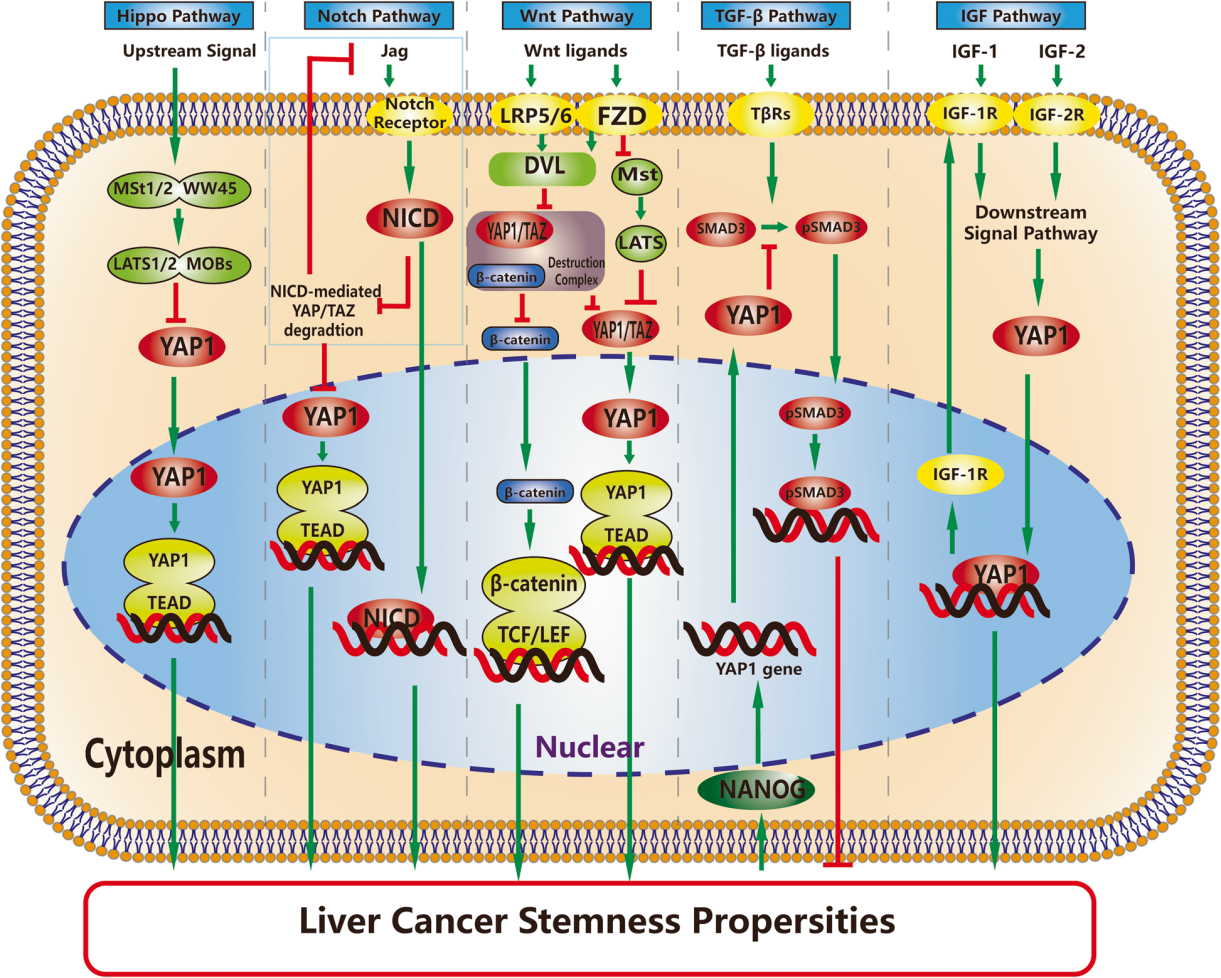
Signaling pathways involved in YAP1-related LCSCs. Several signaling pathways listed above have been found to be involved in the promotion of YAP1-induced LCSCs. Among them, the Notch pathway and IGF pathway are affected by YAP1 in turn by positive feedback loops. The interactions between these pathways also play roles in YAP1-induced LCSCs, and the Wnt pathway suppresses the YAP1-Notch positive feedback loop and upstream components in the Hippo cascade

In addition to the Hippo pathway, YAP1/TAZ activities are also regulated by Hippo-independent pathways, including mechanotransduction, metabolic routes and signaling pathways such as the Wnt/β-catenin pathway. These pathways regulate the nuclear translocation of YAP1/TAZ [16]. This section will focus on the influence of several important YAP1-related signaling pathways in LCSCs (Fig. 2).

#### Hippo pathway

The Hippo tumor suppressor signaling pathway is a highly conserved pathway that restricts organ size and proliferation and has emerged in many types of cancers, including hepatocellular carcinoma [11]. The disturbance of its components caused by pathological conditions will naturally lead to the occurrence of liver cancer and CSCs [17] by affecting YAP1. MOB-deficient liver cells lead to YAP1 overexpression and mediate tumor initiation, which manifests as hyperproliferation, hepatocyte dedifferentiation, overexpression of transforming growth factor beta (TGF-β)2/3, enhanced EMT and cell migration [18]. Similarly, by elevating LATS2 mRNA expression and subsequently downregulating nuclear YAP1, the RNA-binding protein dead end 1 (Dnd1) could inhibit EMT and LCSC prosperities [19].

In HCC, the knockdown of TAZ inhibits cell growth under normal conditions but induces compensatory upregulation of YAP1 together with an increase in the expression of the CSC marker CD90 in the presence of 5-FU, which contributes to chemoresistance [20]. The YAP1-TEAD interaction also plays an essential role in inducing LCSCs. The expression of YAP1 and stemness markers increases as the malignancy of HCC increases. However, in mutant HCCs (YAP1-S94A/S127A) where YAP1 cannot interact with TEAD, the expression of stemness markers did not increase, while YAP1 expression increased [14]. Similarly, hepatocyte 4α (HNF4α) could inhibit the YAP1-TEAD combination and thereby suppress tumorsphere formation of LCSCs without inhibiting YAP1 expression [21].

The Hippo pathway is regarded as the most important signaling pathway regulating the expression of YAP1. From the studies mentioned above, it is clear that the upstream components of YAP1, including MOBs and LATS, its paralog TAZ, and the combination of YAP1 and TEAD could regulate cancer stemness by affecting the expression of YAP1.

#### TGF-β pathway

Regulating cell growth, apoptosis, differentiation and fibrosis, the TGF-β pathway is instrumental in mammalian development and tumor suppression through inhibition of proliferation and induction of apoptosis [22]. After the association of TGF-β ligands and their receptors the TGFβ receptor types I and II (TβRI and TβRII), TβRs form a complex and phosphorylate R-SMADs (SMAD2/3). Then, activated R-SMADs form a complex with SMAD4 and consequently translocate into the nucleus, bind to DNA and regulate the expression of target genes [23]. A study showed that a defective TGF-β tumor suppressor pathway contributes to various malignancies, including HCC, but interestingly, activated TGF-β signaling can induce plasticity toward a more mesenchymal state in HCC cell models. This finding implies that TGF-β plays a complex role in the plasticity and stemness of HCCs [24].

The interaction between TGF-β and YAP1 is also complex. TGF-β1 suppresses the growth of HCC by inducing the upregulation of LATS1 and nucleocytoplasmic translocation and degradation of YAP1 [25], while positive crosstalk between the TGF-β–SMAD and YAP1/TAZ pathways has also been observed. Specifically, YAP1 activation increases the transcription of TGF-βs, and activated TGF-βs increase the nuclear translocation of YAP1 by activating SMAD2/3 [18].

YAP1-induced liver cancer stemness is also implicated in the TGF-β signaling pathway. YAP1 has been identified as a NANOG target gene [26], which exerts its oncogenic activities via suppression of the TGF-β-SMAD3 pathway. Specifically, it has been found in CD133^+^ NANOG^+^ LCSCs that NANOG is induced by ectopic upregulation of TLR4, which can bind to the NANOG site in the proximal promoter region of YAP1 and thereby induce the expression of YAP1. Overexpression of YAP1 then inhibits the TGF-β signaling pathway by suppressing SMAD3 phosphoactivation and p-SMAD3 nuclear translocation. Defective TGF-β signaling caused by YAP1 promotes stemness, oncogenic activity, and chemoresistance of LCSCs [26]. Evidence has shown that the TGF-β pathway is involved in YAP1-induced LCSCs; however, both its effect on the expression of YAP1 and the role that TGF-β plays in LCSCs are complex. There are still unclear mechanisms needing further study.

#### Wnt pathway

The Wnt/β-catenin signaling pathway is involved in cancer stem cell renewal, cell proliferation and differentiation. As observed in various LCSC models, such as CD133^+^ and EpCAM^+^ LCSCs, Wnt/β-catenin signaling pathway activity supports the hyperproliferation of HCC cells and more progenitor cell-like LCSC characteristics [2]. When Wnt ligands bind to their receptors, which consist of frizzled proteins (FZD) and LRP5/6, the cytoplasmic protein dishevelled (DVL) is activated and suppresses the β-catenin destruction complex. β-Catenin translocates into the nucleus and binds to T-cell-specific factor (TCF)/lymphoid enhancer-binding factor (LEF) transcription factors and consequently leads to target gene transcription [27].

The Wnt/β-Catenin pathway is a signaling pathway that integrates with Hippo signaling. Phosphorylated YAP1 binds directly to β-catenin in the cytoplasm and prevents its nuclear translocation, resulting in suppression of the Wnt/β-catenin pathway. Conversely, β-catenin upregulates YAP1 by binding to the DNA enhancer element of YAP1, and knockdown of β-catenin leads to decreased YAP1 expression. Several layers of complex crosstalk exist between YAP1 and the Wnt/β-catenin pathway [28]. Moreover, the Wnt/β-catenin pathway has been found to be involved in regulating YAP1 for the maintenance and expansion of breast CSCs [29].

A recent study showed that YAP1/TAZ is an indispensable component of the β-catenin destruction complex in Wnt-off cells. YAP1/TAZ is essential for inactivating β-catenin and recruiting beta-transducin repeat-containing protein (β-TrCP), an E3 ubiquitin ligase that can mediate the degradation of YAP1/TAZ and β-catenin. When Wnt signaling is activated, YAP1/TAZ are released from the complex and accumulate in the nucleus to activate oncogenic pathways for cancer progression. Meanwhile, nuclear accumulation of β-catenin increases [10, 30, 31]. In addition, the Wnt pathway represses Notch/YAP1/YAZ positive feedback and suppresses the initiation and progression of HCC [32]. Specifically, activation of the Wnt pathway promotes the nuclear translocation of dimerization partner 1 (DP1), which is able to suppress Notch activity.

FZD2 is a key transducer of the noncanonical Wnt pathway, and a recent study found that FZD2-induced EMT enhances the stem properties and tumorigenesis of HCC by activating YAP1 expression and suppressing LATS and MST1/2 [33]. This study provides a promising correlation between YAP1, the noncanonical Wnt pathway and LCSCs.

R-spondin 2 (RSPO 2) is a secreted regulator of Wnt signaling that functions in development and promotes tissue stem cell renewal. RSPO2 is overexpressed in HCC and promotes the activation of Wnt signaling and Hippo-YAP1 signaling. YAP1 overexpression, as well as Wnt overexpression, is required for RSPO2-induced tumor initiation and progression [34].

HNF4α has been found to suppress the Wnt-β-catenin pathway, thereby leading to the inhibition of EMT and CSC generation [35]. A recent study revealed a double-negative feedback mechanism between YAP1-TEAD and HNF4α expression; HNF4α competes with YAP1 for binding to TEAD4 and thereby suppresses the stemness of LCSCs [21]. This finding suggests that HNF4α might be a potential target for YAP1-induced LCSC treatment.

Recent studies have found that stearoyl-CoA desaturase (SCD), which is related to liver metabolic diseases, could regulate the activation of Wnt and YAP1 signaling and thereby regulate EMT, tumorigenesis and stemness of CSCs via the β-catenin destruction complex. The Wnt-SCD-Lrp5/6 positive loop was found in hepatic stellate cells and LCSCs and contributes to fibrosis and tumor development [36]. Given that there are several layers of cross-regulation between Wnt, YAP1/TAZ and SCD, the interaction of YAP1 and this loop might contribute to the stemness of LCSCs.

In summary, the Wnt pathway is highly linked with stemness and YAP1 expression in many aspects. However, several mechanisms of Wnt/YAP1-induced cancer stemness have not yet been demonstrated in LCSCs. For example, it’s still unclear whether YAP1 plays a role in Wnt-SCD-Lrp5/6-induced LCSCs. A gap remains for further studies.

#### Notch pathway

The Notch pathway is another pathway that has been found in LCSCs. In the Notch pathway, the direct binding of Notch ligands (Jag and Delta-like) and their receptors activates the Notch pathway and consequently induces sequential proteolytic cleavage of Notch receptors to generate the Notch intracellular domain (NICD), which enters the nucleus to participate in the transcriptional regulation of target genes [37]. The activation level of Notch is in line with the clinical severity and prognosis of HCC, and downregulation of Notch signaling leads to downregulation of the self-renewal properties of LCSCs, suggesting that Notch signaling contributes to the stemness of LCSCs [38].

Research has found that positive feedback exists between Notch signaling and YAP1/TAZ promoting severe hepatomegaly and rapid HCC initiation and progression [32]. Notch signaling enhances YAP1/TAZ activity by inhibiting TAZ protein degradation through NICD, and YAP1/TAZ can activate Notch signaling by activating the expression of Jag1. Moreover, overexpression of YAP1/TAZ induced by Notch signaling was found to promote HCC initiation and the expression of the stem markers SOX9 and EpCAM, suggesting that the positive feedback between YAP1/TAZ and the Notch pathway may contribute to LCSCs. Notably, this study also found that the Wnt pathway could suppress this positive feedback by controlling DP1 nuclear localization [32].

#### IGF pathway

The insulin-like growth factor (IGF) pathway is a signaling pathway involved in HCC [39]. The core components of the IGF pathway include IGF-1, IGF-2 and their receptors IGF-1 receptor (IGF-1R) and IGF-2R. The binding of IGF ligands and receptors could stimulate downstream pathways and thereby mediate cell processes [40].

Sorafenib is a unique effective therapy for advanced HCC, and sorafenib-acquired resistant tumors show significant stemness, specifically sphere formation in vitro, tumorigenesis in vivo and the expression of stemness markers. YAP1-modulated CSCs are closely correlated with sorafenib resistance in HCC [15]. A recent study found that the acquired sorafenib resistance of HCC is induced by LCSCs via activation of the IGF pathway [41]. The expression level of YAP1, as well as IGF-1 and IGF-1R, was significantly higher in sorafenib-resistant HCCs. Furthermore, it has been found in sorafenib-resistant HCC that YAP1 can induce an increase in EMT and high expression of IGF-1R, both of which are highly related to cancer stemness. Furthermore, activation of IGF-1R induced by IGF treatment promotes the expression and nuclear translocation of YAP1 [42]. In other words, a YAP1-IGF-1R signaling loop exists that plays a role in the sorafenib resistance and cancer stemness of HCC.

### Microenvironment

**Fig. 3:**
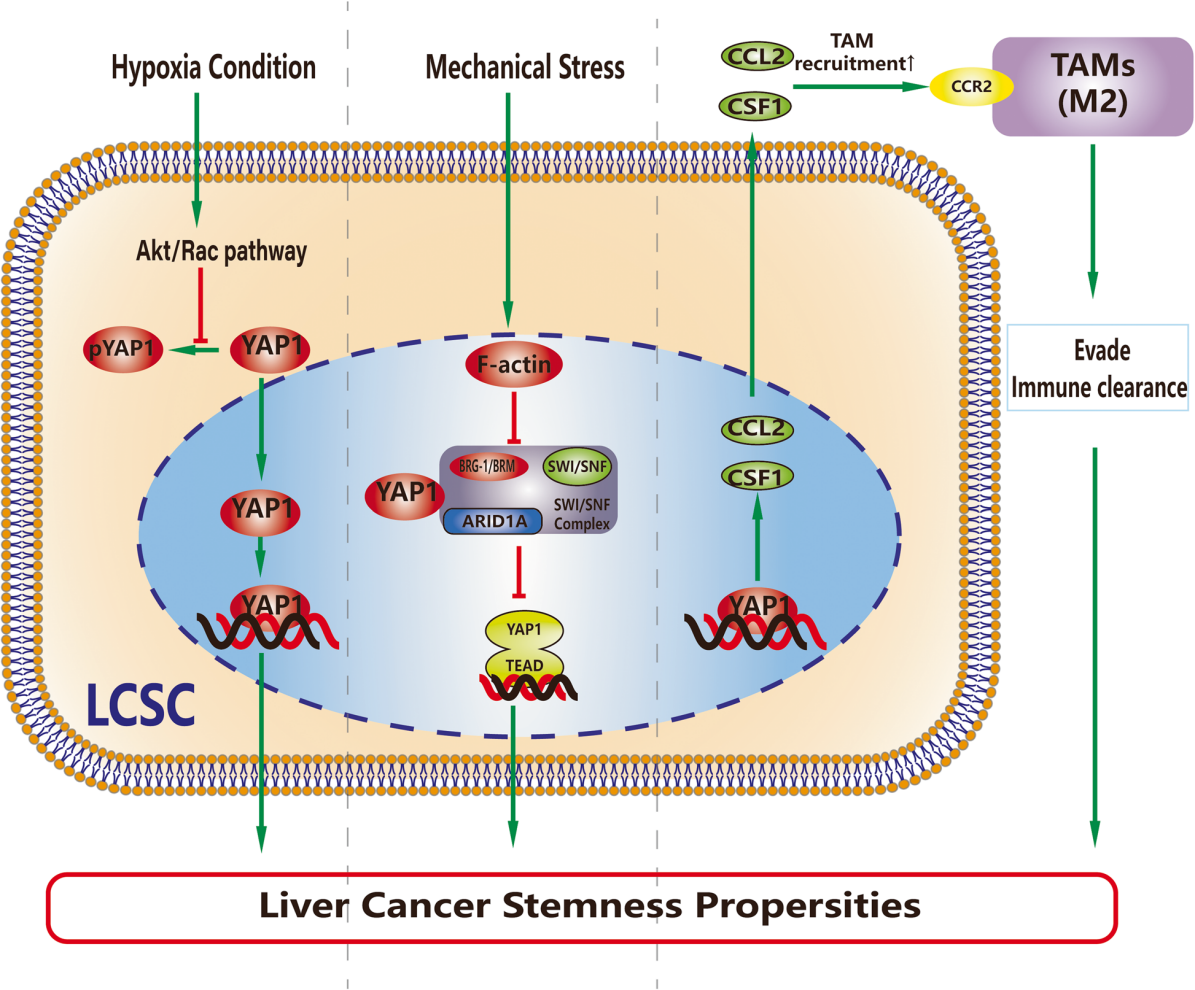
Physical and cellular factors in the microenvironment involved in YAP1-induced promotion of LCSCs. Physical factors, including hypoxic conditions and a high cell density, have been found to promote liver cancer stemness by activating YAP1. TAM recruitment by YAP1 overexpression also leads to the promotion of LCSCs. For CAFs, although they are directly related to the progression of LCSCs and YAP1 overexpression, further studies are needed to determine whether there is a causal relationship between the three. In addition, whether there are other mechanisms of mechanical stress-mediated YAP1-induced promotion of LCSCs remains to be explored

The tumor microenvironment (TME) in HCC comprises the extracellular matrix (ECM), which contains a large variety of cells other than liver cancer cells and is believed to contribute to cancer stemness (Fig. 3). The TME is considered one of the important elements for cancer progression and therapeutic resistance.

### Physical factors

Mechanotransduction enables cells to perceive and adapt to external forces and physical constraints. According to studies, both high and low ECM stiffness contribute to promoting cancer stemness [43, 44]. High ECM stiffness activates the Akt/mTOR signaling pathway in an integrin β1-dependent manner to promote cancer stemness. As the stiffness increased, HCC cells exhibited greater stemness [43]. A recent study [45] demonstrated that YAP1 might play a role in high matrix stiffness-induced LCSCs. As mechanotransducers, YAP1 and TAZ can perceive mechanical signals exerted by ECM rigidity and cell shape via Rho GTPase activity and reorganization of the cytoskeleton. Notably, this process is independent of the Hippo/LATS cascade [46].

Nuclear YAP1/TAZ can form a complex with AT-rich interacting domain-containing protein 1A (ARID1A), Brahma homolog (BRM), Brahma-related gene (BRG-1) and SWItch/Sucrose Non-Fermenting (SWI/SNF). A previous study found that this SWI/SNF complex, which is also known as the BAF (BRGI/BRM-associated factor) complex, could regulate the maintenance of LCSCs by activating the Wnt pathway.

A study suggested that mechanical strain enhances the initiation of liver CSC self-renewal, and this process may be related to lncBRM-mediated YAP1 signaling, which is also independent of the Hippo/LATS cascade [47]. However, the specific mechanism is still unclear. Another recent study discovered that the SWI/SNF complex could bind to YAP1/TAZ directly and inhibit its interaction with TEAD. This association is mediated by ARID1A, and such inhibition is predominant in cells when mechanical stress, which was expressed as cell density in this study, is low. At high mechanical stress levels, nuclear F-actin binds to ARID1A–SWI/SNF, thereby preventing the formation of the ARID1A-SWI/SNF-YAP1/TAZ complex; the YAP1/TAZ-TEAD combination is therefore activated, and stemness properties are induced [48]. Interestingly, it is fairly obvious that this pathway regulates stemness properties by inhibiting the binding of YAP1 and TEAD in the nucleus, which means that this axis is as independent of the Hippo/LATS cascade as the LncBRM-SWI/SNF complex-YAP1 axis. The considerable overlap between these two axes and the fact that binding of YAP1 to BRM/BRG-1 requires ARID1A may indicate a certain connection between these two axes.

Hypoxic conditions induce the expression of EMT-related genes and the stemness markers CD44 and Oct4. Mesenchymal stem cells (MSCs), a major compartment of the TME, have been shown to promote HCC progression through YAP1-mediated lipogenesis reprogramming in hypoxic conditions [49]. In addition, hypoxia contributes to the chemoresistance of HCC by enhancing the accumulation and nuclear translocation of YAP1. A recent study found that Akt/Rac pathway activation induced by hypoxic conditions could increase the expression of Fasin-1, thereby leading to the activation of the Hippo-YAP1 pathway. In this way, hypoxic conditions in HCC result in the promotion of stemness properties [50]. Interestingly, this hypoxia-induced process is not significantly associated with the hypoxia-inducible factor-1 (HIF-1) pathway.

The effect of physical influences on YAP1-induced LCSCs is relatively weak. In fact, as the research mentioned above shows, physical factors, such as hypoxic conditions, ECM stiffness and cell density, contribute to CSCs and the expression of YAP1. However, the mechanisms of YAP1-induced LCSCs promoted by physical influences have not yet been fully demonstrated. The gap in this research field remains to be filled with more studies.

### Cellular components

Cellular components in the microenvironment, including cancer-associated fibroblasts (CAFs) and tumor-associated macrophages (TAMs), are related to LCSCs. Studies indicate that HCC cells with cancer stemness properties might cooperate with macrophages to facilitate tumor initiation. Conditioned medium from polarized tumor-infiltrated type II (M2) TAMs promotes tumor growth, migration, tumorigenesis and stemness by secreting chemokines [51]. Furthermore, the Hippo pathway could suppress the growth and tumor formation of HCC by inhibiting YAP1 and thereby downregulating TAMs in the microenvironment [52]. Moreover, it has been demonstrated that LCSCs can actively evade immune clearance via YAP1-related TAM recruitment even from the single-cell stage. Playing a key role in this process, YAP1 could directly recruit M2 macrophages by stimulating the expression of chemokine ligand 2 (CCL2) and colony stimulating factor 1 (CSF1) for liver carcinogenesis [53]. Interestingly, although both CCL2 and CSF1 are target genes of YAP1, CCL2 is a direct target gene of YAP1-TEAD, while CSF1 is not significantly affected by the association of YAP1-TEAD. It is also notable that although the recruitment effect of YAP1 depends on the interaction of CCL2 and its receptor CCR2 on TAMs, other CCR2+ cells have the opposite effect, which is tumor suppression [53]. In addition, ovatodiolide, which suppresses YAP1-modulated cancer stem cell phenotypes in highly malignant HCC [15], could prevent the polarization of M2 TAMs through YAP1 oncogenic pathways [54].

Previous studies have demonstrated that CAFs increase the tumorigenicity of HCC cells. Conditioned medium from CAFs promotes sphere formation and the expression of stemness-related genes. Except for the CAFs in HCC, peritumoral fibroblasts could potentially recruit and maintain CSCs. Normal fibroblasts are usually quiescent, but when their intrinsic signaling pathway is abnormal, they are induced to differentiate into CAFs [55, 56]. The function of YAP1 is critical for the formation of CAFs and the maintenance of their own characteristics. The YAP1/TEAD1 protein complex has the capability to influence downstream cytoskeletal proteins by regulating SRC transcription and converting normal fibroblasts (NFs) to CAFs in the TME in this way [55, 56]. This suggests that the YAP1/TEAD complex contributes to the maintenance and progression of CSCs by converting NFs to CAFs, but the mechanism still needs further study.

The TME, especially the tumor immune microenvironment, is becoming a popular direction in tumor research. Considering that YAP1 is associated with many aspects of the microenvironment in both normal physiological conditions, HCC and even LCSCs, it is reasonable to infer that there might be more unknown mechanisms between YAP1-induced LCSCs and the TME.

### Epigenetic factors

#### MicroRNAs and lncRNAs

YAP1-induced HCC is closely related to noncoding RNA. LncBRM, together with YAP1 targets, is highly expressed in HCC cells, and the expression level increases with tumor severity. It has been demonstrated that lncBRM can sequester BRM and switch BRM/BRG-1 in the SWI/SNF complex. The BRG1-embedded SWI/SNF complex triggers activation of YAP1 signaling in LCSCs. In this way, the lncBRM-YAP1 axis modulates the self-renewal of LCSCs and is involved in tumor initiation [47].

YAP1 is the direct target of miRNA375. A study found that the expression of miR-375 could diminish the transcriptional activity of YAP1 and suppress endogenous YAP1 protein levels, leading to inhibition of the proliferation and invasion of HCC cells [57]. LncRNA MALAT1, as a ceRNA, can modulate the stemness of LCSCs by sponging miR375 to regulate the post-transcriptional expression level of YAP1 [58].

Adriamycin (ADR), also known as doxorubicin, is a first-line chemotherapy agent for TACE. HCC patients with a poor response to TACE have higher YAP1 expression. Moreover, studies have found that YAP1 plays a crucial role in ADR-resistant HCC by promoting stemness and ATP-binding cassette (ABC) transporters in ADR-R cells. Specifically, YAP1 is targeted by miR590-5p directly, and the miR590-5p-YAP1 axis plays a major role in ADR-resistant HCC [59]. The YAP1-mediated chemoresistant phenotype was closely related to increased expression of stemness markers and ABC transporters. In addition, lncRNA KCNQ1OT1 was found to be able to regulate the self-renewal capacity of LCSCs by targeting YAP1, but the mechanism remains unclear.

Binding of YAP1 and TEAD could directly induce miR-130a, which could suppress VGLL4, an inhibitor of YAP1 activity, by competing for TEAD binding. In this way, miR-130a enhances YAP1 signaling. The YAP1-miR-130a-VGLL4 positive feedback loop mediates hepatomegaly and liver tumorigenesis [60]. However, whether this positive feedback loop involves LCSCs is still unclear. Similarly, miR-132 could lead to tumor regression of HCC by targeting YAP1 and suppressing the expression of YAP1 [61]. Several kinds of noncoding RNAs have been found to regulate the stemness of LCSCs by targeting YAP1 directly or indirectly (Table 1). It is clear that epigenetic modulation plays an essential role in YAP1-induced LCSCs.

**Table 1:** Noncoding RNAs play roles in LCSCs by regulating YAP1

Noncoding RNA	Mechanism	Reference
LncBRM	Switching SWI/SNF complex and thereby activating YAP1	[47]
MiRNA375	Regulating YAP1 expression at a post-transcriptional level directly	[58]
LncMALAT1	Sponging miRNA375 as ceRNA	[58]
MiR590-5p	Directly regulating YAP1 in ADR-R HCC	[59]

#### Epigenetic modulation

Epigenetic modulation, including DNA methylation, histone modification and noncoding RNA, is closely linked to LCSCs [2]. A study showed that histone deacetylase (HDAC) could upregulate the expression of stemness markers and promote cell growth and self-renewal of LCSCs via histone modification [62]. In addition, identified HDAC inhibitors, such as LBH589, JNJ26481585, LAQ824 and SAHA, were demonstrated to suppress YAP1 expression and specifically target the viability and growth of YAP1-induced tumors [63]. Notably, the HDAC inhibitor SAHA also suppressed the stemness of LCSCs. Combined treatment with two kinds of drugs, namely, the histone deacetylase inhibitory activity of valproic acid (VPA) and simvastatin (SIM), could inhibit the maintenance and progression of CSCs in prostate cancer by inhibiting YAP1 [13]. Taken together, these results suggest that HDAC might contribute to the acquisition of liver cancer stemness by increasing the expression and function of YAP1.

### Metabolism

Metabolic reprogramming is viewed as an epiphenomenon of malignant transformation and a crucial driving force promoting the progression and metastasis of cancer cells. Recent studies have found that the plasticity of metabolic reprogramming could enable CSCs to modify their replicative capabilities according to specific needs. The effect of metabolic reprogramming on cancer stem cells via regulation of YAP1 has been demonstrated in lung cancer and CRC. The cancer stem marker NANOG in LCSCs can maintain the stemness of LCSCs by regulating mitochondrial metabolism reprogramming [64]. However, whether metabolic reprogramming contributes to the stemness of LCSCs by regulating YAP1 still needs further research.

#### Lipid metabolism

Stearoyl-CoA desaturase 1 (SCD1) converts saturated fatty acids into monounsaturated fatty acids (MUFAs). SCD1 plays a crucial role in lipid metabolism is closely related to metabolic diseases such as diabetes and fatty liver diseases. SCD1 can activate the Wnt pathway and release β-catenin and YAP1 from the β-catenin destruction complex, thereby promoting the stemness of CSCs by increasing YAP1 nuclear translocation [65]. Considering the Wnt-SCD positive feedback found in HCC [36] and the fact that SCD promotes stemness of HCC via ER stress, SCD might promote LCSCs by activating YAP1.

The mevalonate pathway (MVP) is an essential pathway in lipid metabolism that is important for the biosynthesis of cholesterol and the activity of small GTPases such as Ras, Rho and Rac [13]. Research has found that by promoting the correct membrane anchoring of Rho, activation of MVP can increase the expression and nuclear translocation of YAP1 and thereby maintain the stemness of CSCs [65]. It has been demonstrated that chemical treatment targeting the MVP-YAP1 axis leads to inhibition of CSCs [13]. Notably, regulation of YAP1 by MVP is independent of LATS1/2 [66].

#### Glucose metabolism

O-GlcNAcylation is a specific type of post-translational modification catalyzed by O-GlcNAc transferase (OGT). Metabolic disease-induced high glucose levels could lead to dysregulation of O-GlcNAcylation. A study found that the levels of OGT and O-GlcNAcylation are increased in HCC. In addition, OGT could promote the stemness of liver cancer cells via O-GlcNAcylation of eukaryotic initiation factor 4E [67]. O-GlcNAcylation was implicated in cancer stem cells in lung cancer, prostate cancer, breast cancer and HCC. O-GlcNAcylation modification of YAP1 suppresses the phosphorylation of YAP1 and enhances the expression, function and stability of YAP1 [67]. The O-GlcNAcylation of YAP1 is essential for the tumorigenesis of high glucose-induced HCC. Notably, YAP1 could increase glucose uptake and global cellular O-GlcNAcylation in turn and thereby establish positive feedback [68]. All of these results suggest that the interaction between O-GlcNAcylation and YAP1 contributes to the stemness of HCC induced by high glucose metabolism diseases such as diabetes.

## Conclusion and discussion

The present review summarizes several aspects of research related to the expression of YAP1 and the stemness of LCSCs. The correlation between YAP1 and the stemness of LCSCs is evidenced by many aspects, such as genetic and epigenetic factors, signaling pathways and the microenvironment. As mentioned above, there have been several kinds of proteins found to be associated with YAP1 and playing roles in YAP1-inducing LCSCs (Table 2). The ultimate goal of researching the relationship between YAP1 and LCSCs is to find effective therapeutics against HCC. Playing central roles in cancer initiation, metastasis, recurrence, and therapeutic resistance, LCSCs have been highlighted in research on HCC therapeutics. There are already several therapeutic strategies [66] targeting the LCSC microenvironment, LCSC surface markers and LCSC-related pathways. YAP1 could also be a therapeutic target to suppress the stemness of LCSCs. In fact, progress has been made in this field. Ovatodiolide could significantly reduce YAP1 expression and subsequently suppress YAP1-modulated CSC phenotypes and HCC progression and increase the sensitivity of HCC cells to sorafenib [15].

**Table 2:** Yap-associated proteins involved in LCSC related mechanisms

Proteins	Mechanism
LATS1/2 MOBs	Phosphorylating YAP1
14–3-3 protein	Leading to cytosolic sequestration of YAP1
TEAD	Interacting with YAP1 and activating downstream gene expression
β-Trcp	Mediating ubiquitination and degradation of YAP1
SMAD	P-YAP1/SMAD interaction inhibiting SMAD3 phosphoactivation
OGT	Mediating O-GlcNAcylation of YAP1
ARID1A/SWI/SNF	Binding to YAP1 and inhibiting its interaction with TEAD
β-catenin	Forming β-catenin destruction complex with YAP1

Chemoresistance is one of the properties of CSCs and a major reason leading to the poor prognosis of HCC. Several studies have demonstrated that YAP1 plays a role in resistance to sorafenib [15, 42], 5-FU [20] and ADR [59] in liver cancer chemotherapy. Moreover, YAP1-mediated chemoresistance could be correlated with the stemness of LCSCs. In other words, by targeting expression or nuclear translocation, we can resensitize chemoresistant HCCs. The combination of canonical treatment and YAP1-targeting treatment could result in a better therapeutic effect on chemoresistant HCC induced by LCSCs.

EMT has been found to be closely related to YAP1-induced LCSCs. Overexpression of YAP1 leads to LCSCs by increasing EMT and IGF-1R [42]. Furthermore, inhibition of YAP1 by Dnd1 results in weakening of EMT, thus reducing the stem properties of HCC [19]. Interestingly, in the induction of LCSCs, YAP1 is not only an active inductor of EMT but also a downstream inducer of EMT. FZD could promote the expression of YAP1 and the stemness of HCC by inducing EMT [33]. In view of these studies and the close relationship between EMT and YAP1, we could further focus on the role of EMT-related factors, such as cell polarity, in YAP1-induced LCSCs.

Cell competition [69] is a notable theory correlated with the role of YAP1 in HCC. Activation of YAP1 and TAZ was found in normal liver cells surrounding HCC. YAP1 and TAZ exert tumor-suppressive functions. Deletion of YAP1 and TAZ in HCC suppresses tumor growth, while deletion of YAP1 and TAZ in peritumoral hepatocytes promotes tumor growth. This study suggests a mechanism by which the relative expression levels of YAP1 and TAZ in tumor cells and peritumoral hepatocytes determine the progression of HCC. Whether this mechanism is related to the stemness of LCSCs remains unclear.

## Data Availability

Not applicable.

## Abbreviations

YAP1: Yes-associated protein 1
HCC: Hepatocellular carcinoma
CSCs: Cancer stem cells
TICs: Tumor-initiating cells
LCSCs: Liver cancer stem cells
Wnt: Wingless
5-FU: 5-fluorouracil
TACE: Transcatheter arterial chemoembolization
TAZ: Transcriptional coactivator with PDZ-binding motif
MST: Mammalian STE20-like protein
LATS: Large tumor suppressor homolog
Sav: Mammalian ortholog of Salvator
MOBs: Mps One Binder Kinase Activator
TEAD: TEA domain family member
TBD: TEAD-binding domain
WW: W-containing domain
CC: Coiled-coil domain
TAD: Transactivation domain
EMT: Epithelial–mesenchymal transition
TGF-β: Transforming growth factor beta
Dnd1: Dead end 1
HNF4α: Hepatocyte nuclear factor 4α
TβRI and TβRII: TGFβ receptor type I and II
FZD: Frizzled proteins
DVL: Dishevelled
TCF: T-cell-specific factor
LEF: Lymphoid enhancer-binding factor
β-TrCP: Beta-transducin repeat-containing protein
DP1: Dimerization partner 1
RSPO 2: R-spondin 2
SCD: Stearoyl-CoA desaturase
NICD: Notch intracellular domain
IGF: Insulin-like growth factor
IGF-1R: IGF-1 receptor
TME: Tumor microenvironment
ECM: Extracellular matrix
ARID1A: Domain-containing protein 1A
BRM: Brahma homolog
BRG-1: Brahma-related gene
SWI/SNF: SWItch/Sucrose Non-Fermenting
BAF: BRGI/BRM-associated factor
MSCs: Mesenchymal stem cells
HIF-1: Hypoxia-inducible factor-1
CAFs: Cancer-associated fibroblasts
TAMs: Tumor-associated macrophages
M2: Polarized tumor infiltrated type II
CCL2: Chemokine ligand 2
CSF1: Colony stimulating factor 1
NF: Normal fibroblast
ADR: Adriamycin
ABC: ATP-binding cassette
HDAC: Histone deacetylase
VPA: Valproic acid
SIM: Simvastatin
MUFAs: Monounsaturated fatty acids
MVP: Mevalonate pathway
OGT: O-GlcNAc transferase
